# Wharton’s jelly mesenchymal stromal cells derived from preterm umbilical cord reveal a hepatogenic potential

**DOI:** 10.3389/fcell.2025.1626353

**Published:** 2025-06-24

**Authors:** F. Timoneri, M. Lo Iacono, S. Corrao, G. Alberti, G. Amico, T. Corsello, P. G. Conaldi, Eleonora Russo, G. La Rocca

**Affiliations:** ^1^ Department of Regenerative Medicine and Immunotherapy - Ri.MED Foundation, Palermo, Italy; ^2^ Department of Biomedicine, Neurosciences and Advanced Diagnostics (BiND), University of Palermo, Palermo, Italy; ^3^ Department of Biological, Chemical and Pharmaceutical Sciences and Technologies (STEBICEF), University of Palermo, Palermo, Italy; ^4^ Department of Pediatrics, Division of Clinical and Experimental Immunology and Infectious Diseases (CEIID), University of Texas Medical Branch, Galveston, TX, United States; ^5^ Research Department, IRCCS ISMETT, (Istituto Mediterraneo per i Trapianti e Terapie ad Alta Specializzazione), Palermo, Italy; ^6^ Departmental Faculty of Medicine, Saint Camillus International University of Health Sciences, Rome, Italy

**Keywords:** Wharton’s jelly, mesenchymal stromal cells, preterm umbilical cord, hepatogenic potential, immune modulation, liver diseases, therapeutic abortion

## Abstract

**Introduction:**

The umbilical cord (UC) is a perinatal tissue from which it is possible to isolate mesenchymal stromal cells (MSCs) with a higher proliferation rate and a higher differentiation capacity compared to their adult counterparts. Wharton’s jelly (WJ) is a rich source of multipotent and hypoimmunogenic MSCs (WJ-MSCs), which are considered promising candidates for cell therapy of many conditions, including liver diseases. Preterm umbilical cord (birth before 37 weeks of gestation) is generally considered a waste product, so its use does not affect ethical issues. Similarly to the full-term UC, it can be considered a valid source of WJ-MSCs, although little is known about their phenotype and differentiation capacity. We aim to show that WJ-MSCs derived from preterm umbilical cords exhibit comparable characteristics to their mature counterparts and, in particular, show a similar ability to differentiate into hepatocyte-like cells and retain hypoimmunogenicity features. Both these aspects may be key in the prospective application of these cells in regenerative medicine.

**Material and Methods:**

Here, we isolated WJ-MSCs from seven prematurely harvested umbilical cords (pWJ-MSCs). We assessed a mesenchymal phenotype and differentiation potential by flow cytometry, immunocytochemistry, and immunofluorescence. A standardized liver differentiation protocol was performed, and the acquisition of the hepatic phenotype was assessed by characterization of hepatic protein expression and functional assays.

**Results:**

We demonstrated that pWJ-MSCs exhibited mesenchymal characteristics, acquired phenotypical and functional features of hepatocytes when induced to differentiate in a specific medium, and maintained the expression of immunomodulatory molecules.

**Discussion:**

This study showed that even if pregnancy has been interrupted before the 22nd week, p-WJ-MSCs had the same differentiative ability as the counterpart derived from the full-term UC, that we have previously demonstrated, and can be considered a valid cellular population for liver cell therapy.

## 1 Introduction

In regenerative medicine, cell therapy with mesenchymal stromal cells (MSCs) has certainly attracted the attention of many researchers and clinicians. MSCs key features are self-renewal, multipotency, and immunomodulation. Depending on the source, age of the donor, and culture conditions, MSCs showed striking differences (Garcia-Bernal et al., 2021). In this regard, numerous studies have focused on gaining a comprehensive knowledge of these cells through *in vitro* and *in vivo* experiments. A careful understanding of MSCs fits better with the choice of cell type specific to different diseases. Many clinical studies showed the success of MSCs transplantation in various conditions, but the mechanisms that contribute to these positive outcomes are far from being fully understood. Researchers believe that therapeutic success is due to the multiple effects of MSCs such as paracrine signaling, immune modulation, and extracellular vesicles release, either with or without a prior differentiation (Liang et al., 2014) Understanding and optimizing these mechanisms may improve MSC-based therapies for conditions such as autoimmune diseases, neurodegenerative disorders, cardiovascular and end-stage liver disease (ESLD). In this regard, liver regeneration can be divided into two categories based on the source of regenerating hepatocytes: hepatocyte-driven regeneration (typical regeneration) and liver progenitor cell-driven regeneration (alternative regeneration) (Li et al., 2021). In cases of extensive hepatocyte loss, alternative regeneration plays a crucial role. Advances in liver regeneration research and tissue engineering have contributed significantly to the development of regenerative medicine strategies for the treatment of ESLD. In this scenario, MSCs represent a promising cell source for the treatment of liver diseases, not only for their hepatogenic differentiation potential but also for their ability to promote liver regeneration and inhibit liver fibrosis (Zhang et al., 2020).

MSCs derived from perinatal tissues have several advantages over MSCs derived from adult sources such as bone marrow and adipose: non-invasive isolation procedure, lack of ethical concerns, better proliferation, and lower immunogenicity (Kern et al., 2006).

The umbilical cord (UC) is considered a reservoir for various cell types, with MSCs making up the largest proportion of cells. The MSCs derived from the WJ (WJ-MSCs) are promising for a variety of applications. According to the criteria of the International Society for Cellular Therapy (Dominici et al., 2006), WJ-MSCs show a mesenchymal phenotype and can differentiate into adipocytes, osteoblasts, and chondrocytes (La Rocca et al., 2013). Various studies have emphasized the ability of WJ-MSCs to differentiate into other cell types such as endothelial cells (Chen et al., 2009), retinal cells (Ozmert and Arslan, 2020), hepatocyte-like cells (Lo Iacono et al., 2025), neuron-like cells (Zhang et al., 2017) and insulin-producing -β cells (Soleimanifar et al., 2024).

Due to their natural location in placental tissue, WJ-MSCs have decisive immunological properties. They express both canonical (HLA-A, HLA-B, HLA-C) and non-canonical (HLA-E, HLA-F, HLA-G) MHC class I antigens, while they lack MHC class II molecules such as HLA-DR, HLA-DP, and HLA-DQ (La Rocca et al., 2013) (Anzalone et al., 2013a). In addition, WJ-MSCs do not express costimulatory antigens important for T and B cell activation, including CD40/CD40L, CD80, CD86, and B7-DC. In addition, WJ-MSCs were found to express B7-H3, a negative regulatory molecule that suppresses T-cell proliferation (Corsello et al., 2019). These cells also inhibit the differentiation and maturation of monocytes into dendritic cells through contact-dependent mechanisms while promoting the development and maturation of regulatory T cells (Tregs). This underlines their robust immunomodulatory capabilities (Vieira et al., 2019). The tissue regeneration capacity of WJ-MSCs is also due to their ability to stimulate the cells on site, which secrete many biological molecules as well as growth factors, cytokines, and interleukins. In this respect, the conditioned medium derived from WJ-MSCs represents a promising alternative to direct cell transplantation and offers a safer, more scalable, and versatile approach for the treatment of various diseases.

Ongoing clinical and preclinical research is aimed at optimizing the WJ-MSC-derived conditioned medium by identifying the most effective bioactive components and establishing standardized production and application protocols (Acuto et al., 2023). Innovations such as bioreactor technology, enrichment with extracellular vesicles, and genetic modification of MSCs have the potential to further improve therapeutic efficacy.

Recently, many researchers have focused on the study of MSCs from perinatal tissues (umbilical cord and amniotic membrane) for the treatment of acute respiratory syndrome and cerebral hemorrhage that can occur in premature infants (Dabrowski et al., 2017; Omar et al., 2022).

The use of this cell type does not imply any alloreaction, as the donor and recipient are identical, rendering the clinical application easier. The relationship between the age of the stem cell source and regenerative properties remains poorly understood (Bustos et al., 2014), so researchers investigated MSCs from preterm and term tissue to determine any differences. In this context, Messerli et al. have shown that WJ MSCs from preterm births have a similar ability to differentiate into neural progenitor cells as those from full-term births (Messerli et al., 2013). In addition, research on hematopoietic progenitor cells (HPCs) suggests that preterm cord blood contains more HPCs that have greater clonogenic potential than those from full-term cord blood (Podesta et al., 2015).

In 2013, Lim et al. compared the efficacy of human amniotic epithelial cells from preterm birth in alleviating inflammation and fibrosis using a rodent model of lung injury (Lim et al., 2013). While preterm cells exhibited higher yield, viability, and proliferative capacity, their ability to reduce inflammation and fibrosis was lower than that of term amniotic cells. Recently, a study investigated the therapeutic potential of cord blood cells from preterm and term infants in a large animal model of white matter injury (Li et al., 2017). Both preterm and term cells successfully normalized white matter density and reduced cell death in sheep exposed to hypoxic-ischemic brain injury. However, the underlying mechanisms differed: premature cells reduced tumor necrosis factor α, whereas term cells upregulated interleukin-10 and attenuated oxidative stress.

Moreira and coworkers emphasized that WJ-MSCs from preterm and term UC had similar characteristics: they expressed CD73, CD90, and CD146, but lacked the expression of CD117, CD79, and HLA-DR, were able to differentiate into three mesodermal cell lineages, and had comparable proliferation rate, cell motility and colony formation efficiency (Balgi-Agarwal et al., 2018).

Another paper evaluated the effects of exposure to different oxygen tension (hypoxia, normoxia, and hyperoxia) in pre-term and term WJ-MSCs: all cells shared similar features relative to proliferation, motility, cell viability, senescence, and inflammatory cytokines expression (Balgi-Agarwal et al., 2018).

Here, we point out that WJ-MSCs derived from preterm UC show comparable features to WJ-MSCs derived from UC at term, but importantly preterm cells, in specific culture conditions, show a hepatogenic potential as demonstrated by hepatocyte markers expression and acquisition of liver functions. For these reasons, preterm WJ-MSCS could be considered valid and alternative tools for the treatment of liver diseases in comparison with adult counterparts.

## 2 Materials and methods

### 2.1 Isolation and *in vitro* expansion of preterm Wharton’s jelly-MSCs (pWJ-MSCs)

The isolation of pWJ-MSCs was performed following our previously published protocol used to isolate WJ-MSCs from term cords (Lo Iacono et al., 2025). Seven human preterm UC (n = 7) were obtained following therapeutic abortion after the mother’s consent according to the tenets of the Declaration of Helsinki and local ethical regulations (IRRB/58/13, Ismett, Institutional Research Review Board). In Supplementary Table S1 are listed the identification code, gestational age, fetal pathology, and cell passage (Supplementary Table S1 **)**.

Briefly, umbilical cords were harvested from therapeutic aborted fetuses at a gestational age between 15 and 22 weeks and aseptically stored at room temperature in Hanks’ balanced salt solution (HBSS, Lonza) supplemented with 20 mL/L antibiotics/antimycotics (A5955, Sigma-Aldrich, Germany) until processing. The isolation procedure was performed within 6 h of the harvest. The preterm cords were cut into small pieces, 1 cm in length, after two washes in the presence of HBSS. Based on the natural migratory ability of mesenchymal cells, a non-enzymatic protocol was conducted (La et al., 2009). To increase the exposure of the WJ to the plastic surface, each piece was longitudinally incised with a sterile scalpel without vessels removal. The pieces were then placed into 6-well plates, with the Jelly substance facing to the plastic surface. The outgrowing pWJ-MSCs were cultured in ‘complete medium’ composed of low glucose Dulbecco’s Modified Eagle’s medium (DMEM) (D5523, Sigma-Aldrich, Germany), supplemented with 10% fetal bovine serum (FBS gold, PAA), 1x NEAA (M7145, non-essential amino acids, Sigma-Aldrich, Germany), 1x antibiotics/antimycotics (A5955, Sigma-Aldrich, Germany), and 2 mM L-glutamine (G7513, Sigma-Aldrich, Germany). The cultures were maintained at 37ºC in a humidified atmosphere at 5% CO2. The medium was changed every second day, and the culture was followed under a light microscope. After 15 days the cord pieces were removed and when the adherent spindle-shaped cells reached the 80% confluence they were detached with Tryple Select Enzyme (12,563, Gibco, United States) and cultured for up to 10 passages. The viability of the isolated cells was routinely evaluated by trypan blue exclusion.

### 2.2 Characterization of pWJ-MSCs by flow cytometry, immunocytochemistry/immunohistochemistry and immunofluorescence analysis

To characterize pWJ-MSCs using flow cytometry (FC), 1 × 10^5^ cells were used at passages 2 and 5 (p2 and p5) for each antibody staining. Isotype control antibodies were used as negative controls for the measurement of the non-specific binding of the primary antibodies. Fixation and permeabilization steps were performed with the Cytofix/Cytoperm™ kit (554,714, BD Biosciences) according to the manufacturer’s instructions. The fluorescein isothiocyanate (FITC)-, phycoerythrin (PE)-, or allophycocyanin (APC)-, Peridinin-Chlorophyll-Protein (PerCP)- conjugated antibodies and isotype-matched controls (BD Biosciences) used for the FC analyses are summarized in Supplementary Table S2. Forward and side scatter gates were set to include all viable cells. Routinely, debris and doublets were excluded from the cell population data by the application of forward and side scatter selection. The co-expression of two markers was analyzed by the gating of the population that was positive for the first marker and the subsequent analysis of the percentages of the second marker. FC data were acquired with a BD FACS Aria II instrument (Becton Dickinson) and were analyzed with FlowJo ^TM10^ version software.

*For immunofluorescence analysis,* the cells were plated in 8 well chamber slides (354,118 BD Falcon, Germany). After reaching 80% confluence, the cells were fixed according to the antibody manufacturer’s instructions. After incubation with a blocking solution composed of PBS with 0.5% Tween20% and 3% BSA (bovine serum albumin), cells were incubated with the primary antibodies overnight at 4°C, rinsed with PBS, and then incubated with the appropriate fluorescence-conjugated secondary antibodies (AlexaFluor, Invitrogen, Carlsbad, CA) for 1 h at room temperature in the dark. Cells were mounted with Prolong Gold Antifade (Invitrogen, Carlsbad, CA), which includes diamidino-2- phenylindole (DAPI) for the nuclear counterstaining and stored in the dark at 4°C until fluorescence microscope analyses. HepG2 cells were used as the positive control for albumin and AFP expression in culture. Cell imaging was done with a microscope (Nikon Eclipse 50i, Melville, NY) coupled with a camera (Olympus XM10, Tokyo, Japan) and Cell F software for image acquisition (Olympus). The primary antibodies are listed in Supplementary Table S3.

*For immunocytochemical analysis*, we operated following our previously published protocols (Lo Iacono et al., 2025). Briefly, cells were plated in 8-well chamber slides (354,118 BD Falcon, Germany), reached the 80% confluence were fixed with cold methanol for 20 min, and permeabilized with PBS with 0.1% TRITON-X100. After washing in PBS slides were exposed to 0.3% H_2_O_2_ in PBS and a blocking solution, composed of serum (provided by Vectastain stain kit). After incubation with specific primary antibodies (listed in Supplementary Table S3) for 1.5 h at room temperature, appropriate secondary antibodies were used. The detection was performed using an ABC peroxidase staining kit (VectorLab) and AEC Substrate-Chromogen (Dako Golstrup, Denmark). The cells were counterstained with hematoxylin to identify the nuclei. Negative controls were used simultaneously by omitting the primary antibody step.

*For immunohistochemistry analysis,* 5-µm human liver sections, derived from archival tissue specimens stored as formalin-fixed paraffin-embedded blocks, were used as a positive control for the analysis of the primary antibodies directed to hepatic-related molecules. The staining was performed by using a streptavidin–peroxidase kit (LSAB2 system peroxidase; Dako, Golstrup, Denmark). Briefly, after deparaffinization steps, the sections were exposed to 0.3% hydrogen peroxide solution for 10 min. After 1 h of incubation with blocking solution (PBS with 3% BSA), specific primary antibodies have been added for staining (listed in Supplementary Table S3). Negative controls were run simultaneously without the addiction of primary antibodies. After several washing steps with 1X phosphate buffer saline (PBS) (Sigma-Aldrich, Germany), sections were incubated with mouse/rabbit universal secondary antibodies from the LSAB2 system kit. After 1 h of incubation, streptavidin–peroxidase followed by 3-amino-9-ethylcarbazole (AEC) (Dako, Golstrup, Denmark) were added and nuclei were counterstained using hematoxylin (Dako, Golstrup, Denmark).

### 2.3 Adipogenic, osteogenic, and chondrogenic differentiation protocols

The differentiation protocols here performed were previously described by our group (La Rocca et al., 2013).

*Adipogenic differentiation* was performed by culturing pWJ-MSCs, at the 5th passage, with adipogenic medium, in which culture “complete medium” was supplemented with 0.5 mM isobutylmethylxanthine (I7018, Sigma-Aldrich, Germany), 1M dexamethasone (I7378, Sigma-Aldrich, Germany)), 10M insulin (Sigma-Aldrich, Germany), 200M indomethacin (Sigma-Aldrich, Germany)), 10% FBS Gold, and 1% antibiotic/antimycotic. Cells were cultured in 6-well tissue culture plates for 3 weeks, and the medium was replaced every second day. The formation of cytoplasmic lipid vacuoles was monitored by phase-contrast microscopy along with culturing. Controls included naïve pWJ-MSCs cultured in a standard growth medium for 3 weeks (named NT pWJ-MSCs). To demonstrate the adipogenic differentiation, cells were stained with Oil Red O (O139, Sigma-Aldrich, Germany). Briefly, pWJ-MSCs control and induced differentiation were fixed with 10% formalin (Sigma-Aldrich, Germany) for 30 min at room temperature, followed by subsequent washes with distilled water and 60% isopropanol. Oil Red O working solution was added to the cells for 5 min. After 4 washes we performed the counterstaining with Meyer’s hematoxylin for 1 min.

*Osteogenic differentiation* was induced on monolayer pWJ-MSCs, at the 5th passage, using a standard “complete medium” supplemented with 50M ascorbate-2-phosphate (Sigma-Aldrich, Germany)), 10 mM β-glycerophosphate (G5422, Sigma-Aldrich, Germany)), and 0.1 µM dexamethasone (D1756, Sigma-Aldrich, Germany). Naïve pWJ-MSCs cultured in a standard growth medium were included as controls (named NT pWJ-MSCs). After 3 weeks, the osteogenic phenotype was assessed using the Alizarin Red S (A5533, Sigma-Aldrich, Germany)). Briefly, cells were fixed in 4% paraformaldehyde and stained with 1% solution of Alizarin Red S.

*Chondrogenic induction* was performed in an alginate beads three-dimensional system as previously described (La Rocca et al., 2013). Before encapsulation, the cells, at the 5th passage, were pelleted by centrifugation and gently resuspended at a cell concentration of 3 × 10^6^ cells/ml, in a 1.2% solution of sterile filtered low-viscosity sodium alginate (Sigma- Aldrich, Germany). The cell suspension was loaded in a sterile syringe and slowly dispensed (drop by drop) in a solution containing 100 mM calcium chloride, causing gelation of the alginate as microspheres containing the cells. Groups of four to five microspheres were then cultured in standard medium or chondrogenic medium (DMEM supplemented with 1% FBS Gold, 6.25 μg/mL insulin, 10 ng/mL transforming growth factor beta-1 (TGF-β1), 50 nM ascorbate-2-phosphate, 1% antibiotic/antimycotic, and 1x NEAAs). After 3 weeks, beads were collected into 50 mL conical tubes and fixed for 4 h at room temperature with 4% paraformaldehyde in 0.1 M sodium cacodylate/10 mM calcium chloride buffer. After fixation, the specimens were rinsed overnight at 4ºC in 50 mM BaCl_2_ (barium chloride) in cacodylate buffer (pH 7.4) to irreversibly cross-link the alginate matrix. Then the beads were dehydrated with a graduated ethanol series, cleared with xylene, and embedded in paraffin. For histological staining, 4-μm sections were cut. Before proceeding with the staining protocol, the sections were deparaffinized and rehydrated. For proteoglycan detection, alcian blue staining (Sigma-Aldrich) was performed using standard procedures (Podesta et al., 2015). Alcian blue working solution (1% Alcian blue 8GX in 3% acetic acid, pH 2.5) was added for 30 min. After washing, nuclei were counterstained in 0.1% nuclear fast red (Sigma-Aldrich) solution. After different differentiation protocols, the cells were observed with an inverted microscope (DMIL, Leica, DMI 300 B).

### 2.4 Hepatocyte-like cells differentiation protocol

pWJ-MSCs were used for hepatic differentiation experiments at the 5th passage. We used as initial inducers two growth factors: fibroblast growth factors (FGFs) and hepatocyte growth factor (HGF) as previously described in (Lo Iacono et al., 2025). Induction of hepatic differentiation was performed with DMEM low glucose, 1% FBS (FBS Gold, PAA), 1x NEAA (M7145, Sigma-Aldrich, Germany), 1x antibiotics/antimycotics (A5955, Sigma-Aldrich, Germany), 2 mM L-glutamine (Sigma-Aldrich, Germany), 10 ng/mL of human FGF-4 (Miltenyi Biotech, Germany), 20 ng/mL of human HGF (Miltenyi Biotech, Germany), 1x Insulin-transferrin-selenite supplement (ITS, I3146, Sigma-Aldrich, Germany), and 0.1 µM dexamethasone (Sigma- Aldrich, Germany). Medium changes were performed each other day. After 3 weeks, the initial hepatic medium was further supplemented with 10 ng/mL of oncostatin M (OSM, Miltenyi Biotec, Germany) for another week. For all the lines tested in differentiation experiments, the control pWJ-MSCs (NT pWJ-MSCs) were cultured in the standard medium as previously described. Both the treated and control pWJ-MSCs were analyzed at the end of the 3rd and 4th week of the hepatic differentiation process. Constant cell monitoring throughout the culture period was performed by phase-contrast microscopy. Treated cells were referred to as pHLCs.

### 2.5 Detection of glycogen with periodic acid schiff (PAS)

PAS staining was performed using a commercial kit (395B, Sigma-Aldrich, Germany) following the manufacturer’s instructions. Briefly, control pWJ-MSCs and pHLCs (both cells at 5th passage), were fixed in formaldehyde/ethanol fixative solution for 1 min. After washing, periodic acid was added for 15 min at room temperature. Following several washes with distilled water, Schiff’s reagent was added for 15 min at room temperature. Subsequently, after another washing, the cells were stained with Gill’s hematoxylin for 90 s. Slides were observed at the light microscope.

### 2.6 Glucose-6-phosphatase (G-6-Pase) activity assay

We used a previously published protocol (Lo Iacono et al., 2025). Briefly, both NT pWJ-MSCs (control cells) and pHLCs (both cells were used at passage 5) were incubated with a working solution composed of 2.4 mM lead nitrate and 2.08 mM glucose-6-phosphate in 0.1 M Tris-acetate buffer pre-warmed at 37°C. After 20 min, the working solution was removed, and after washing, 5% ammonium sulfide was added for 30 s to convert lead nitrate into lead sulfate, which is visible as brownish–black precipitates. Hematoxylin was used to counterstain and after we observed cells with an inverted microscope (DMIL, Leica, DMI 300 B).

### 2.7 Indocyanine green uptake assay

Cardiogreen or indocyanine green (21,980, Sigma-Aldrich) is a dye soluble in water. This compound can form non-covalent fluorescent complexes with some plasma proteins, mainly with albumin. Indocyanine green linked with albumin is rapidly taken up by the hepatocytes and then excreted unchanged into bile. Briefly, confluent NT pWJ-MSCs and pHLCs (at passage 5) were incubated for 15 min at 37ºC with 1 mg/mL of cardiogreen in a culture medium. After three washes with PBS, cells were observed by microscope.

### 2.8 Detection of CYP450 3A4 metabolic activity

To evaluate CYP4503A4 activity, we used a kit that is labeled P450-GloTM CYP3A4 Assay (Luc-PFBE, Promega, United States) and followed the manufacturer’s instructions. To summarize, NT pWJ-MSCs and pHLCs (at 5th passage) were stimulated with 25 µM rifampicin (Sigma-Aldrich, Germany). The cells were simultaneously incubated with 10 µM ketoconazole, an inhibitor of CYP4503A4 (Sigma-Aldrich, Germany) for 48 h. Daily medium changes were made. After removing the dirt with PBS, the cells were exposed to 50 µM of luciferin-PFBE for 4 h in the darkness. After addiction of 50 µL luciferin detection and incubation at room temperature for 20 min, the samples were read by a luminometer (Promega, United States) using integration times of 0.5 s. A luminogenic substrate was added to the medium without cells, to determine background luminescence.

### 2.9 Statistical analysis

Statistical analyses were performed on triplicate samples using GraphPad Prism 4 software (GraphPad Software, San Diego, United States). The significance of differences between the experimental conditions was assessed by the Mann-Whitney test. Values were considered significant for p < 0.05.

## 3 Results

### 3.1 Morphological and phenotypical features of pre-term WJ-MSCs

**FIGURE 1:**
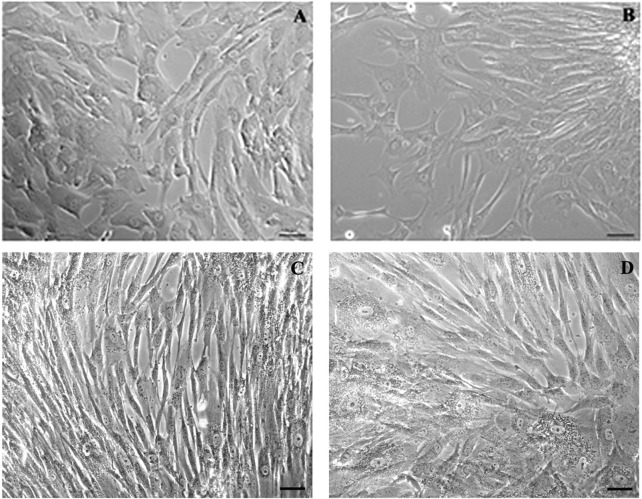
Phase-contrast microscopy micrographs of p-WJ-MSCs in monolayer culture. Micrographs **(A,B)** show WJ-MSCs isolated from umbilical cords at term at 1st and 10th passage, respectively, while **(C,D)** show p-WJ-MSCs at same passages. Magnification: 20x. Scale bar: 25 μm.

**FIGURE 2:**
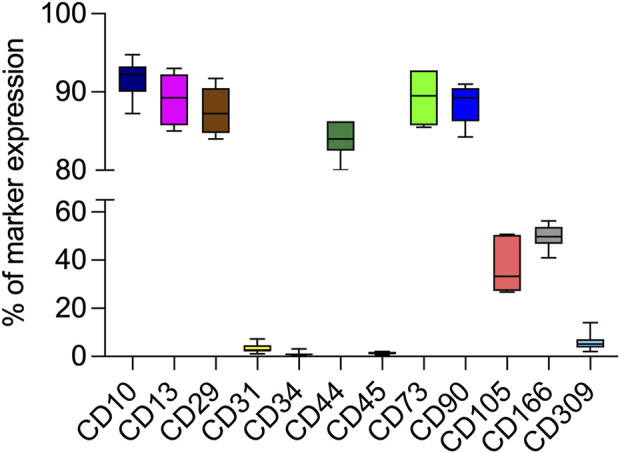
Percentage of CD10, CD13, CD29, CD44, CD73, CD90, CD105, CD166, CD309 expression in p-WJ-MSCs at 2nd and 5th passage by flow cytometry analysis. Data are represented as media ± SD of all 7 p-WJ-MSCs samples. Experiments were performed in duplicate for each passage of culture.

**FIGURE 3:**
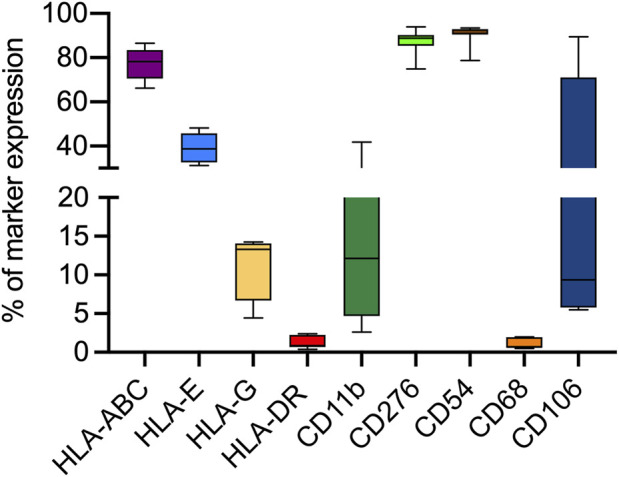
Flow cytometry analysis for the expression of different immune-related and immunomodulatory molecules in all 7 p-WJ-MSCs at different passage (2nd and 5th). Data are represented as media ± SD of all 7 p-WJ-MSCs samples. Experiments were performed in duplicate for each passage of culture.

From 7 preterm umbilical cords, we were able to reproducibly obtain a mesenchymal cell population. Pre-term Wharton’s Jelly-derived mesenchymal stromal cells (pWJ-MSCs) were able to proliferate until the 10th passage, maintaining a fibroblastoid morphology, similar to cells obtained from full-term cords (Figure 1). Flow cytometry analysis confirmed that the pWJ-MSCs showed similar expression of CD10, CD13, CD29, CD44, CD73, and CD90 at all passages, while lower expression was observed for CD105 and CD166 (Figure 2). The pWJ-MSCs were negative for typical hematopoietic and endothelial markers (CD31, CD34, CD45, CD309) (Figure 2). To show that the pWJ-MSCs showed characteristics comparable to counterparts derived from the UC at term, we examined the expression of some immune-related and immunomodulatory molecules responsible for low immunogenicity. As shown by FC analysis (Figure 3), naïve pWJ-MSCs expressed high levels of HLA-ABC in both the 2nd and 5th passages, while expression of HLA-DR was absent. We also observed the expression of two non-classical class I HLAs, HLA-G and HLA-E, both involved in immunomodulation at the feto-maternal interface. The results obtained showed a weak expression of HLA-G. Very interesting was the high expression of CD276 (also known as B7-H3), which plays a role in mediating local interactions of the fetal trophoblast with maternal immune cells at the feto-maternal interface (Kshirsagar et al., 2012) (Figure 3). We also detected in pWJ-MSCs that the other two molecules (CD54 and CD106) are involved in immune regulation and specifically in T cell migration (Melendez et al., 2003) (Figure 3). Moreover, we observed the absence or the extremely low expression of typical monocyte markers such as CD68 and CD11b (Figure 3). All the tested immunomodulatory molecules maintained a stable expression during passages in culture without any appreciable changes. Moreover, we also investigated the expression of molecules belonging to the B7 family, B7-1 (CD80) and B7-2 (CD86), two co-stimulatory molecules required for effector T-cell induction. Neither CD80/B7-1 nor CD86/B7-2 were detectable in any of our pWJ-MSCs (Supplementary Figure S1), thus confirming the expected phenotype for UC-derived cells.

**FIGURE 4:**
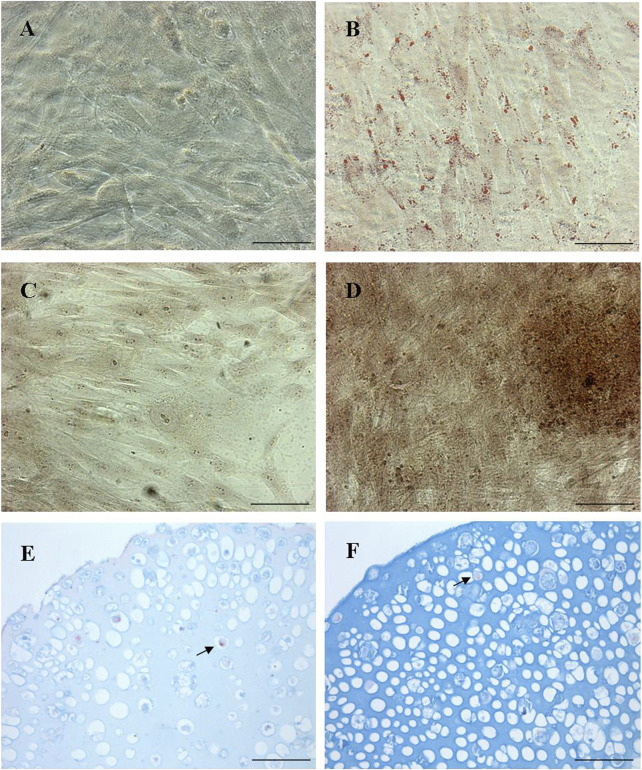
Representative micrographs panel of the tri-lineage differentiation by p-WJ-MSCs at 5th passage. Oil Red O staining in naïve p-WJ-MSCs (control cells) **(A)** and adipocyte-like cells **(B)** after adipogenic differentiation. Alizarin Red S staining in control p-WJ-MSCs **(C)** and osteoblast-like cells **(D)** after osteogenic differentiation. Alcian blu staining in control p-WJ-MSCs **(E)** and chondrocytes-like cells **(F)** Magnification: 20x. Scale bar: 50 µm.

**FIGURE 5:**
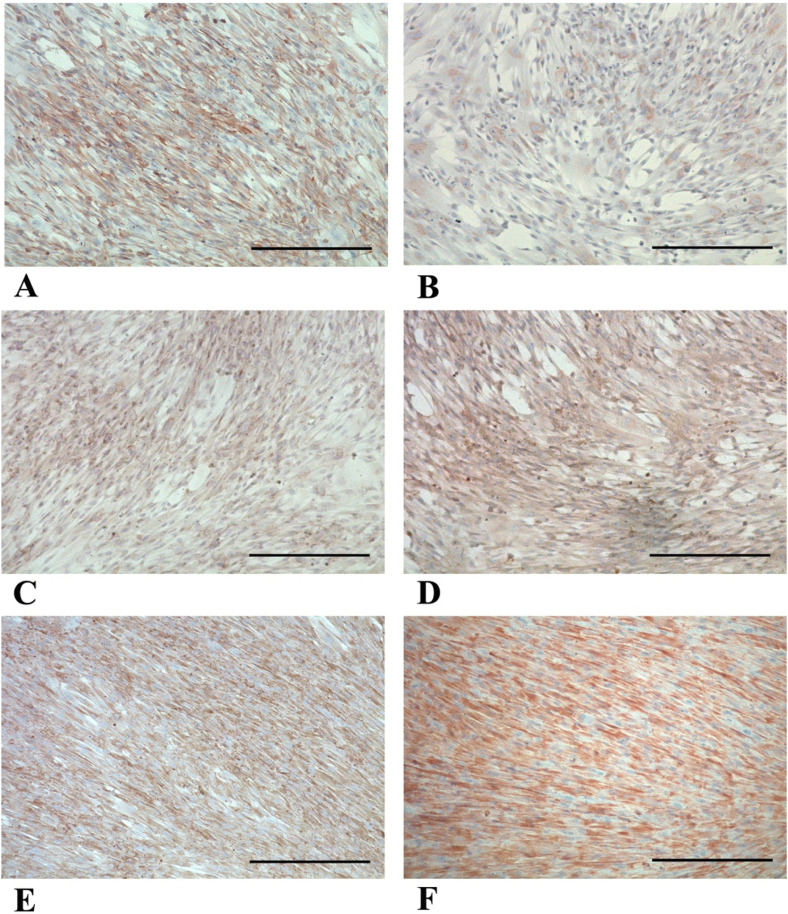
Representative panels of immunocytochemical detection of multipotency markers in p-WJ-MSCs at 5th passage. Micrographs show the expression of Nestin **(A)**, c-KIT/CD117 **(B)**, GATA-4 **(C)**, Connexin-43 **(D)**, CK-8 **(E)** and CK-18 **(F)**. Magnification: 10x. Scale bar:200 µm.

We demonstrated that pWJ-MSCs could differentiate in all three cell types of mesodermal cell lineages: adipocytes, osteocytes, and chondrocytes (Figure 4). Naïve pWJ-MSCs showed adipocyte features following adipogenic differentiation, confirmed by the presence of intracellular lipid droplets, highlighted by Oil Red O staining (Figure 4B). The presence of extracellular calcium deposits detectable in bone nodules-like structures in pWJ-MSCs has been assessed by Alizarin Red S staining (Figure 4D). Moreover, pWJ-MSCs cultured in a chondrogenic medium, in the presence of an alginate matrix, appeared located in rounded lacunae surrounded by a glycosaminoglycan-enriched matrix as demonstrated by Alcian blue staining (Figure 4F), contrarily to control cells (Figure 4E). To confirm the multipotency of pWJ-MSCs, we evaluated the expression of different tissue markers, suggesting their plasticity. In this regard, in Figure 5, we highlighted the expression of two proteins such as nestin (Figure 5A) and c-KIT (known also as CD117) (Figure 5B). The nestin is typically expressed in hepatic, pancreatic, and embryonic progenitor cells (Bernal and Arranz, 2018). CD117/c-Kit is a marker that identifies an undifferentiated cell population (Suphanantachat et al., 2014) that we identified both in pWJ-MSCs and in other adult MSCs populations (Anzalone et al., 2013a). We also identified the expression of GATA-4 (Figure 5C), a zinc finger transcription factor with a key role during liver development (He et al., 2023). In particular, the positivity for GATA-4 in the cytoplasm and at the perinuclear level suggested an inactive state in our undifferentiated pWJ-MSCs (Figure 5; Supplementary Figure S2). Importantly, we also, detected the expression of connexin-43 (Figure 5D), cytokeratin-8 (Figure 5E), and −18 (Figure 5F), all proteins specific to hepatocytes, suggesting that these cells can differentiate towards hepatic cell lineage (Figure 5; Supplementary Figure S2).

### 3.2 Acquisition of hepatogenic phenotype by pWJ-MSCs

**FIGURE 6:**
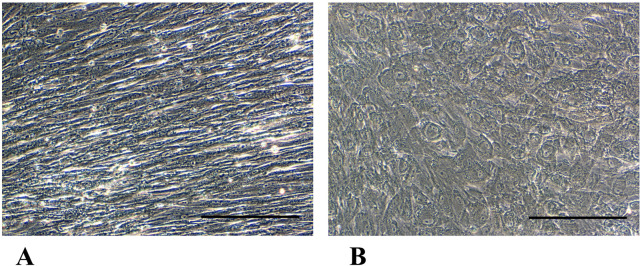
Phase-contrast micrographs panels show morphological changes in p-HLCs **(B)** after 4th week of hepatic differentiation in comparison with control p-WJ-MSCs (NT-p-WJ-MSCs), grown in a standard growth medium for the same time of culture **(A)**. Magnification 20x. Scale bar: 200 µm.

**FIGURE 7:**
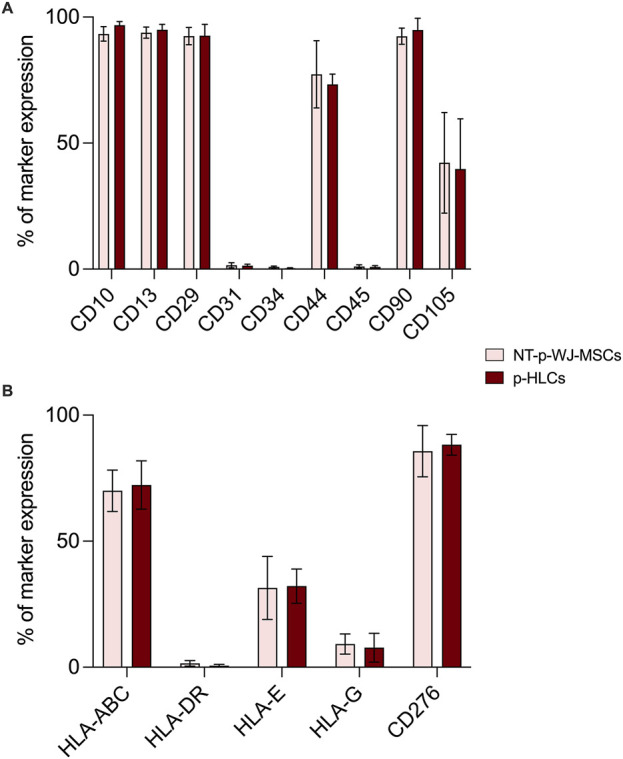
Representative flow cytometry analysis of mesenchymal **(A)** and immunomodulatory molecules **(B)** in NT p-WJ-MSCs and p-HLCs at the 4th week of hepatic differentiation. Data are represented as mean ± SD of n = 4 of NT-p-WJ-MSCs and p-HLCs. Comparison among control (NT-p-WJ-MSCs) and treated cells (p-HLCs) were performed using the Mann-Whitney test. Statistical analysis among two groups resulted statistically non-significant.

Hepatic differentiation was achieved by applying a two-step protocol developed by our group (Lo Iacono et al., 2025), consisting of a “pre-inductive step” and a “maturation step” that mimics *in vitro* the time-dependent signaling events that take place during fetal liver morphogenesis. After 3 weeks of induction, pHLCs (pre-term -hepatocyte-like cells) already showed a morphological transition, from a typical fibroblastic morphology to a polygonal-like shape that closely resembles that of a hepatocyte. This morphological switch was maintained at the 4th week of differentiation (Figure 6). Importantly, we highlighted that the hepatic differentiation protocol did not alter the intrinsic mesenchymal features. In this regard, in Figure 7, we showed that pHLCs maintained the same phenotype of control pWJ-MSCs (named here as NT-WJ-MSCs) (Figure 7A). Moreover, our pHLCs maintained the same expression pattern of immunomodulatory molecules featured before the differentiation (Figure 7B), suggesting that these differentiated cells could be transplanted without host reaction, thanks to their low immunogenicity.

**FIGURE 8:**
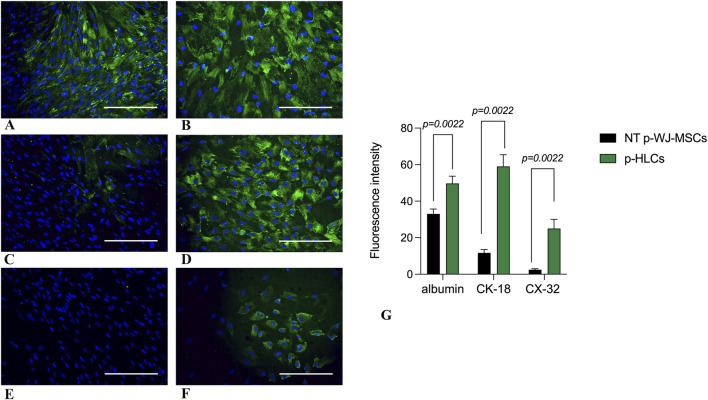
Representative micrographs panel of immunofluorescence detection of hepatic markers in control p-WJ-MSCs and p-HLCs at 4th week of hepatic differentiation. The left panel shows the expression of albumin, cytokeratin-18 (CK-18), and connexin-32 (CX-32) mostly visible in p-HLCs **(B,D,F)** in comparison with control cells **(A,C,E)**. Albumin **(A,B)**; CK-18 **(C,D)**; connexin- 32 **(E,F)**. The right panel **(G)** shows fluorescence intensity expression of Albumin, CK-18, and CX-32 in p-HLCs, at the 4th week of differentiation, in comparison with NT-p-WJ-MSCs (control cells). Data are mean ± SD of n = 4 of NT p-WJ-MSCs and p-HLC. Statistical analysis was performed by Mann-Whitney test. Magnification: 20x. Scale bar = 100 µm.

**FIGURE 9:**
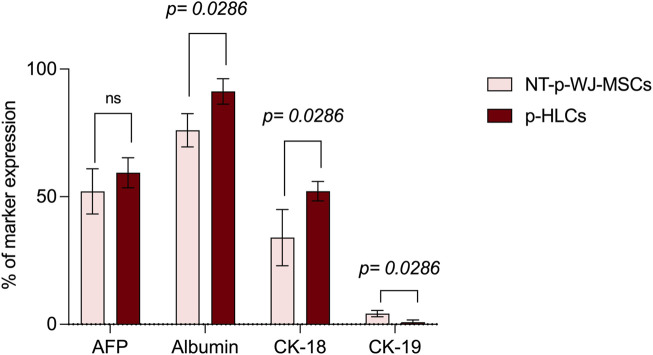
Flow cytometry analysis of early and late hepatic protein expression in NT-pWJ-MSCs and p-HLCs at 4th week of differentiation. Data are represented as mean ± SD of n = 4 NT p-WJ-MSCs and p-HLCs. Statistical analysis was performed by Mann-Whitney test; *ns,* not significant.

The acquisition of hepatic phenotype was confirmed by the presence of mature hepatic specific proteins as well as albumin, CK-18, and Connexin 32 (Cx-32). Liver sections were used as a positive control for the expression of these hepatic proteins (Supplementary Figure S3). In particular, the immunofluorescence analysis highlighted an increase of albumin, CK-18, and Cx-32 expression in pHLCs concerning NT pWJ-MSCs. (Figure 8). These results have also been confirmed by flow cytometry analysis (Figure 9). Moreover, we observed that pHLCs after 4th week of hepatic differentiation lacked the expression of CK-19, a typical cholangiocyte marker (Figure 9). As demonstrated by flow cytometry analysis the expression of AFP was not statistically different between control and differentiated cells, probably because p-WJ-MSCs, being of fetal origin, express at higher level fetal proteins. Importantly, we showed that albumin and AFP expression in HepG2 cells was similar to that detected in our p-HLCs (Supplementary Figure S4 **).** These data may suggest an active maturation process toward a hepatocyte-like phenotype.

### 3.3 Functional assays demonstrate the acquisition of hepatic phenotype by pWJ-MSCs

**FIGURE 10:**
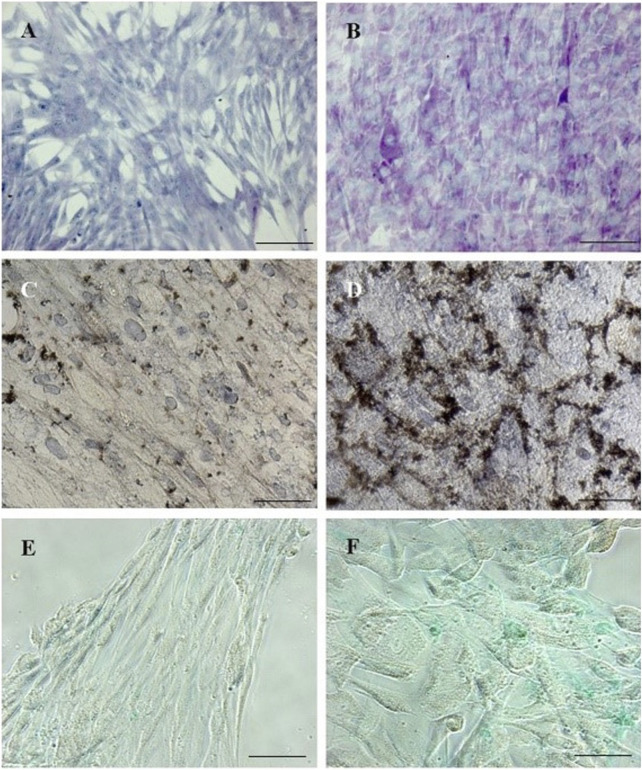
Micrographs panels detecting different mature hepatocyte functions in p-HLCs and NT-WJ-MSCs after 4th week of hepatic differentiation. PAS staining in control p-WJ-MSCs **(A)** showed a pale blue staining, while p-HLCs **(B)** showed a polygonal shape and an intense purple-magenta stain. G-6-Pase assay in NT p-WJ-MSCs **(C)** failed to form precipitates of lead sulphide, while p-HLCs **(D)** exhibited a dark-brown precipitates demonstrating the enzyme activity. ICG uptake experiments demonstrated that p-HLCs **(F)** did uptake mostly the molecule with respect to NT p-WJ-MSCs **(E)**. Magnification: 10x **(A,B)** and 20x **(C–F)**. Scale bar: 100 µm **(A,B)** and 50 µm **(C–F)**.

To further ascertain the acquisition of hepatocyte features, we evaluated the glycogen synthase activity resulting in glycogen deposition by PAS staining. Interestingly, our pHLCs, already at the 3rd week of hepatic differentiation showed the presence of glycogen deposits, and the latter was more visible in pHLCs (Figures 10A,B) during the 4th week of hepatic differentiation.

**FIGURE 11:**
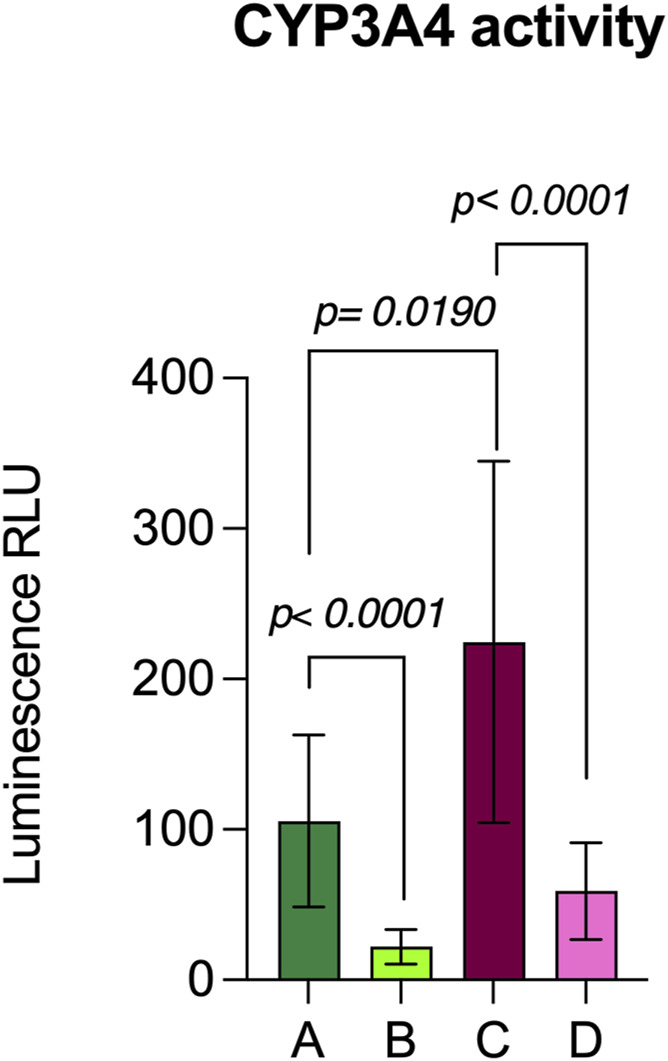
Evaluation of CYP3A4 metabolic activity in NT p-WJ-MSCs and HLCs at 4th week of differentiation. NT p-WJ-MSCs **(A)** and p-HLCs **(C)** in presence of rifampicin, an inducer of CYP3A4 activity. NT p-WJ-MSCs **(B)** and p-HLCs **(D)** in presence of rifampicin but with ketoconazole addiction, an inhibitor of CYP3A4 activity. Data are represented as mean ± SD of n = 4 p-WJ-MSC populations. Statistical analysis was performed by Mann-Whitney test.

We also investigated the activity of another hepatic enzyme, glucose-6-phosphatase (G-6-Pase), involved in glucose metabolism. The presence of G-6-Pase has been revealed in pHLCs at all time points of hepatic differentiation while remaining absent in controls. In Figures 10C,D is possible to observe the presence of sulfate precipitation, from dark-brown staining, only in pHLC concerning NT pWJ-MSCs at 4th week differentiation. The hepatocytes are cells that can selectively uptake molecules. To demonstrate this ability, we performed the indocyanine green assay. We observed an intense cytoplasmatic green staining in pHLC contrarily to control pWJ-MSCS (Figures 10E,F). Since the liver represents the main site of xenobiotic metabolism, we assessed CYP3A4 activity, one of the main cytochromeP450 isoforms, both in NT pWJ-MSCs and pHLCs after 4 weeks of hepatic differentiation. We observed that the CYP3A4 activity increased in the presence of rifampicin in pHLCs rather than in NT pWJ-MSCs, with a significant statistically difference (Figure 11) Importantly the activity of the enzyme decreased following the addition of ketoconazole, a specific inhibitor of CYP3A4, thus confirming its specificity (Figure 11). These data suggest that pHLCs acquire the ability to metabolize xenobiotics in an inducible manner.

## 4 Discussion

WJ-MSCs can originate from either preterm (pWJ-MSCs) or term (WJ-MSCs) births. While both cell populations share common mesenchymal stromal cell properties, literature reports suggest that they exhibit differences in their biological characteristics, proliferation, differentiation potential, and therapeutic applications. In this regard, Iwatani and co-workers observed that the phenotype of pWJ-MSCs and term WJ-MSCs was similar, as well as their ability to differentiate in three cell mesodermal lineages (adipocytes, osteoblasts, and chondrocytes) (Iwatani et al., 2017). On the contrary, by analyzing the gene expression profile, the authors highlighted some differences among the two WJ-MSC cell populations. They observed a significantly higher proliferation rate, by involvement of WNT signaling, in pWJ-MSCs in comparison with term WJ-MSCs (Iwatani et al., 2017). Another study showed that pWJ-MSCs not only have a more primitive phenotype allowing a greater plasticity but could secrete more anti-inflammatory cytokines (IL-10, TGF-b), and show a more vigorous repair activity (Huang et al., 2023). All these data suggest that pWJ-MSCs, similarly to WJ-MSCs obtained from the umbilical cord at the time of birth, could be useful for cell therapy to treat various diseases as well as liver, neurodegenerative and autoimmune diseases. In the context of liver disease, stem cell differentiation has recently been recognized as an alternative to hepatocyte transplantation (Furuta et al., 2020). According to recent literature, perinatal MSCs may represent promising populations to be used for the treatment of end-stage liver diseases, with the ability to repopulate the organ provide mature hepatocyte functions, possibly re-activating local hepatic progenitors, and survive the attacks of the host immune or inflammatory defenses (Iacono et al., 2013) (Anzalone et al., 2013b). We have recently shown that WJ-MSCs derived from term umbilical cord can differentiate into hepatocyte-like cells, acquiring the functional capabilities typical of mature hepatocytes and retaining their immunomodulatory activity (Lo Iacono et al., 2025). Specifically, we showed that after hepatic differentiation, WJ-MSCs express mature liver proteins as well as albumin, CK-8, CK-18 and connexin-32, while lack the expression of CK-19, a cytokeratin specific for cholangioblasts. Moreover, we highlighted the expression of several enzymes involved in cholesterol, lipid, bile acid and alcohol metabolism, detoxification of xenobiotics, liver morphogenesis and/or regeneration, and the urea cycle.

According to previous results, in this study we wanted to assess if pWJ-MSCs, derived from therapeutically aborted fetuses, could show the same intrinsic mesenchymal features of the counterpart at term, and acquire a hepatic phenotype after the same hepatic differentiation protocol (Lo Iacono et al., 2025). We successfully isolated p-WJ-MSCs using a standardized enzyme-free isolation protocol (La et al., 2009), and we displayed that these cells, similarly to mature WJ-MSCs, showed the same morphology (see Figure 1), mesenchymal phenotype (Figures 2, 3), and tri-lineage differentiative ability (Figure 4). Among the identified proteins in naïve pWJ-MSCs, we highlighted the expression of GATA-4, a transcription factor involved during liver development (He et al., 2023), previously also detected in mature counterparts (Lo Iacono et al., 2025). In literature is known that GATA-4-null embryos displayed severe defects in the visceral endoderm, and malformations in the ventral foregut, from which it originates the liver, ventral pancreas, and lungs (Rojas et al., 2010). Moreover, it was demonstrated that the presence of GATA-4 is very important to avoid liver fibrosis (Rojas et al., 2010). Our data also show that naive pWJ-MSCs have the ability to differentiate in many cytotypes, and the expression of nestin, and CD117/c-KIT confirms that these cells can be considered progenitor cells (Wiese et al., 2004). Specifically, the expression of CK-8, CK18, and Connexin-43, reveled in pWJ-MSCs, predicts a potential hepatic differentiation capacity by pWJ-MSCs (shown in Figure 5).

Since immune modulation is one of the most promising aspects of WJ-MSCs use in cell therapy (La Rocca et al., 2013), we verified the expression of HLA-ABC, HLA-DR, HLA-E, HLA-G, and CD276/B7-H3, in pWJ-MSCs. As showed in Figure 3, we observed the lack of HLA-DR expression and the presence of normal levels of class I MHC, such as HLA-A, -B, and–C. These data highlighted that pWJ-MSC have the same immune phenotype concerning to the mature WJ-MSCs (La Rocca et al., 2013; Lo Iacono et al., 2025), and for this reason they may favor the escape of cells from the immune surveillance of the host, increasing the probability of successful engraftment. Moreover, similarly to mature counterparts, pWJ-MSCs express both HLA-E and HLA-G molecules, playing a central role in embryos and placenta, by modulation the immune response of the mother toward the semi-allogeneic fetus. Nevertheless, the lack of CD80/B7-1 and CD86/B7-2 expression in immature WJ-MSCs (see Supplementary Figure S1) is a significant observation because it implies that any residual engagement of the T cell receptor on Th cells would result in anergy and contribute to tolerance rather than allogeneic responses (La Rocca et al., 2012). Another important result is the expression of CD276/B7-H3 molecule, which has been postulated to exert immunoprotected and tolerogenic functions, attenuating peripheral immune responses through co-inhibition (Vigdorovich et al., 2013). Here, we highlighted CD276 expression in pWJ-MSCs, as previously demonstrated also in mature WJ-MSCs (Lo Iacono et al., 2025), suggesting that the presence of this molecule is a common feature of umbilical cord mesenchymal stromal cells independently of cord age. In this study, for the first time, we demonstrated that pWJ-MSCs could successfully differentiate in hepatocyte-like cells (pHLCs), similarly to mature counterparts. As showed in Figures 8, 9, pHLCs, after 4^th^ week of hepatic differentiation, could express mature hepatic proteins as well as CK-8, CK-18, albumin, and connexin-32. We also highlighted the presence of glucose-6-phospatase, glucose-6-phosphatase dehydrogenase and CYP3A4, enzymes involved in gluconeogenesis, glycogenolysis process and xenobiotic metabolism, as previously demonstrated also for mature WJ-MSCs. In addition, we evaluated in pHLCs, the capacity to internalize the cardiogreen, a hepatocyte-specific ability that we here assessed for the first time in cord-derived MSCs (Lo Iacono et al., 2025).

One of the most attractive features of WJ-MSCs is their hypoimmunogenicity. Studies demonstrated that these cells could inhibit *in vitro* allogeneic lymphocyte proliferation (Corsello et al., 2019). Moreover, *in vivo* experiments, tolerogenic functions have been demonstrated following the infusion of WJ-MSCs into immunocompetent hosts (Nauta and Fibbe, 2007; Deuse et al., 2011). These properties have been linked to the absence of class II MHC molecules expression and the capacity to synthesize and secret molecules interfering with the immune system at various levels. Therefore, in this study, we wanted to evaluate the immunomodulatory molecule expression by pWJ-MSCs after hepatic differentiation. Our data demonstrated for the first time that pHLCs maintained the expression of HLA-A, -B, and–C, HLA-E, HLA-G, and more interesting CD276/B7-H3 expression. This immune phenotype, associated with the lack of HLA-DR expression, confers hypoimmunogenicity and tolerogenicity features to the pHLCs, similar to naïve cells (see Figure 7), and WJ-MSCs derived from the umbilical cord at term (Lo Iacono et al., 2025).

In conclusion our results demonstrate that WJ-MSCs, derived from the preterm human umbilical cord, generally considered as a waste tissue, may represent a new source of multipotent MSCs with a significant hypoimmunogenecity. Moreover, exhibiting a hepatogenic potential, pWJ-MSCs can be considered promising tools for the treatment of liver diseases, even if further studies are needed to optimize their therapeutic efficacy in liver disease models.

## Data Availability

The original contributions presented in the study are included in the article/Supplementary Material, further inquiries can be directed to the corresponding authors.
