# Supplementary material for: Wharton’s jelly mesenchymal stromal cells derived from preterm umbilical cord reveal a hepatogenic potential
Source: Front Cell Dev Biol. 2025 Jun 24;13:1626353. doi: 10.3389/fcell.2025.1626353 (PMC12235173; doi:10.3389/fcell.2025.1626353)
Supplement: Supplementary file 1 [file DataSheet1.docx]

Supplementary Material

Supplementary Table 1: Wharton’s Jelly mesenchyamal stromal cells derived from preterm umbilical cord

| **Sample code** | **Gestational age** | **Fetal pathology** |
| --- | --- | --- |
| **WJ-MES 11_13** | 21 weeks | Trisomy 21 |
| **WJ-MES 11_14** | 21 weeks | Multiple Pathologies |
| **WJ-MES 11_16** | 15 weeks | Trisomy 21 |
| **WJ-MES 11_17** | 20 weeks | Spina Bifida |
| **WJ-MES 12_01** | 21,5 weeks | Spina Bifida |
| **WJ-MES 12_02** | 22 weeks | Bilateral ventriculomegaly |
| **WJ-MES 12_03** | 16 weeks | Cardiac insufficiency |

Supplementary Table S2: List of antibodies used for flow cytometry analysis

| Antigen | Clone | Conjugated | Dilution | Manufacturer |
| --- | --- | --- | --- | --- |
| Albumin | 188835 | uncon-jugated | 1:400 | R&D Systems |
| AFP | 189506 | unconjugated | 1:800 | Cedarlane |
| CD10 | HI10a | APC | 1:20 | Becton Dickinson |
| CD13 | WM15 | APC | 1:20 | Becton Dickinson |
| CD11b | ICRF44 | PE-CY7 | 1:20 | Becton Dickinson |
| CD29 | MAR4 | APC | 1:20 | Becton Dickinson |
| CD31 | WM59 | FITC | 1:20 | Becton Dickinson |
| CD34 | 581 | FITC | 1:20 | Becton Dickinson |
| CD44 | G44-26 | APC | 1:20 | Becton Dickinson |
| CD45 | 2D1 | PerCP | 1:20 | Becton Dickinson |
| CD54 | HA58 | APC | 1:20 | Becton Dickinson |
| CD68 | Y1/82A | PE | 1:20 | Becton Dickinson |
| CD71 | M-A712 | APC | 1:20 | Becton Dickinson |
| CD73 | AD2 | APC | 1:220 | Miltenyi Biotec |
| CD90 | 5 E10 | FITC | 1:400 | Becton Dickinson |
| CD105 | SN6 | FITC | 1:20 | Abcam, Cambridge |
| CD106 | 51-10C9 | APC | 1:20 | Becton Dickinson |
| CD117 | YB5.B8 | PE | 1:20 | Becton Dickinson |
| CD166 | 3A6 | PE | 1:20 | Becton Dickinson |
| CD276 (B7-H3) | FM276 | APC | 1:220 | Miltenyi Biotec |
| CD309 | 89106 | APC | 1:20 | Becton Dickinson |
| CK18 | C-04 | FITC | 1:400 | Abcam |
| CK19 | RCK108 | PE | 1:400 | Santa Cruz Biotechnology |
| HLA ABC | W6/32 | FITC | 1/20 | Abcam Cambridge, MA |
| HLA DR | L243 | PerCP | 1:20 | Becton Dickinson |
| HLA-G | 87G | PerCP | 1:20 | eBioscience Inc |
| HLA-E | 3D12HLA-E | APC | 1:20 | eBioscience |
| IGG1 | G18-145 | FITC | 1:20 | Becton Dickinson Biosciences |
| IgG2 | PC10 | PE | 1:20 | Becton Dickinson |

Supplementary Table S3: List of antibodies used for immunofluorescence, immunocytochemistry and immunohistochemistry (*).

| Antigen | Host | Manufacturer | Dilution (IF) | Dilution (ICC) |
| --- | --- | --- | --- | --- |
| Albumin * | Mouse | Santa Cruz | 1:100 | 1:300 |
| AFP | Mouse | Santa Cruz | 1:100 | N/A |
| B7-1  B7-2 | Mouse | Santa Cruz | N/A | 1:800 |
| Connexin 32* | Mouse | Santa Cruz | 1:100 | 1:100 |
| Connexin 43 | Rabbit | Santa Cruz | 1:50 | 1:100 |
| Connexin 45 | Rabbit | Santa Cruz | 1:50 | N/A |
| Cytokeratin 8 | Mouse | Sigma | N/A | 1:200 |
| Cytokeratin 18 * | Mouse | Sigma | 1:800 | 1:800 |
| c-Kit (Cd117) | Rabbit | Epitomics | N/A | 1:50 |
| GATA-4 | Rabbit | Santa Cruz | 1:100 | 1:100 |
| Nestin | Mouse | Santa Cruz | 1:100 | 1:100 |

N/A: data not available


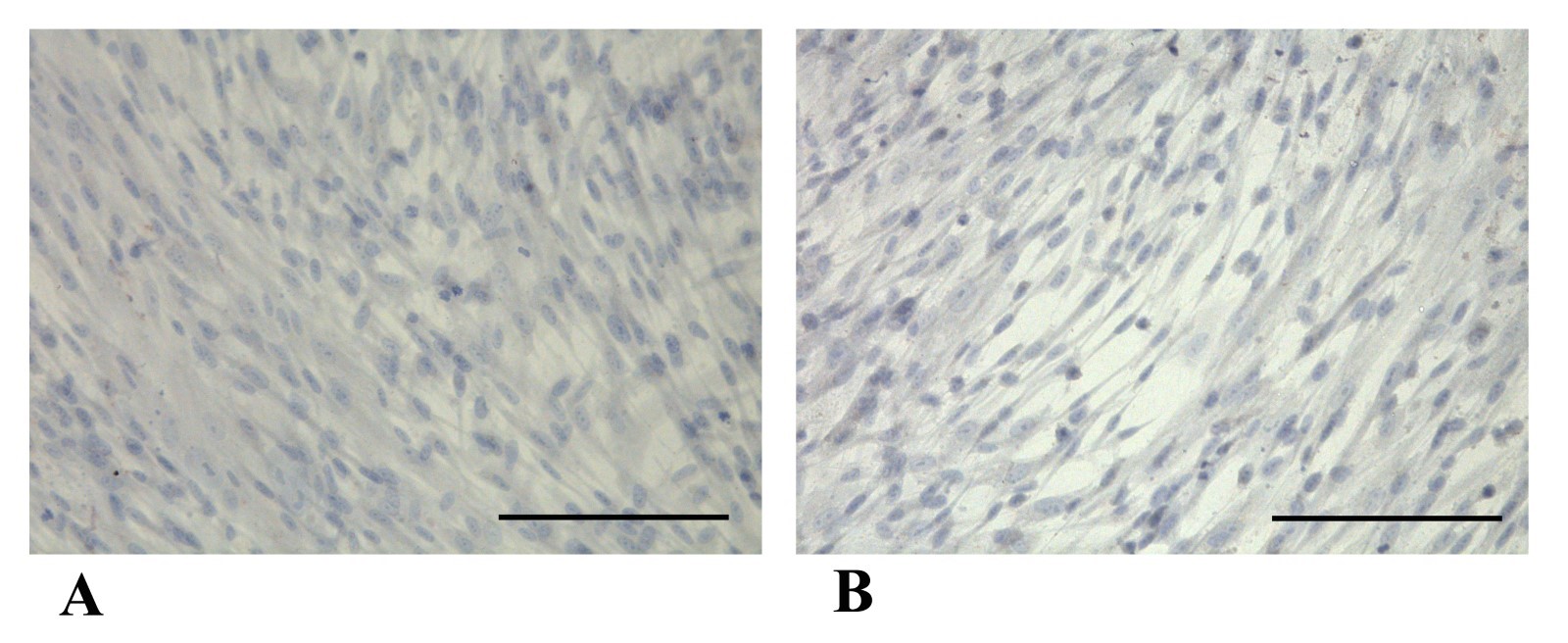


Supplementary Figure S1: Representative micrographs showing the immunocytochemical analysis of CD80 (A) and CD86 (B)in p-WJ-MSCs. Magnification: 20x.; Scale bar: 100 µm.


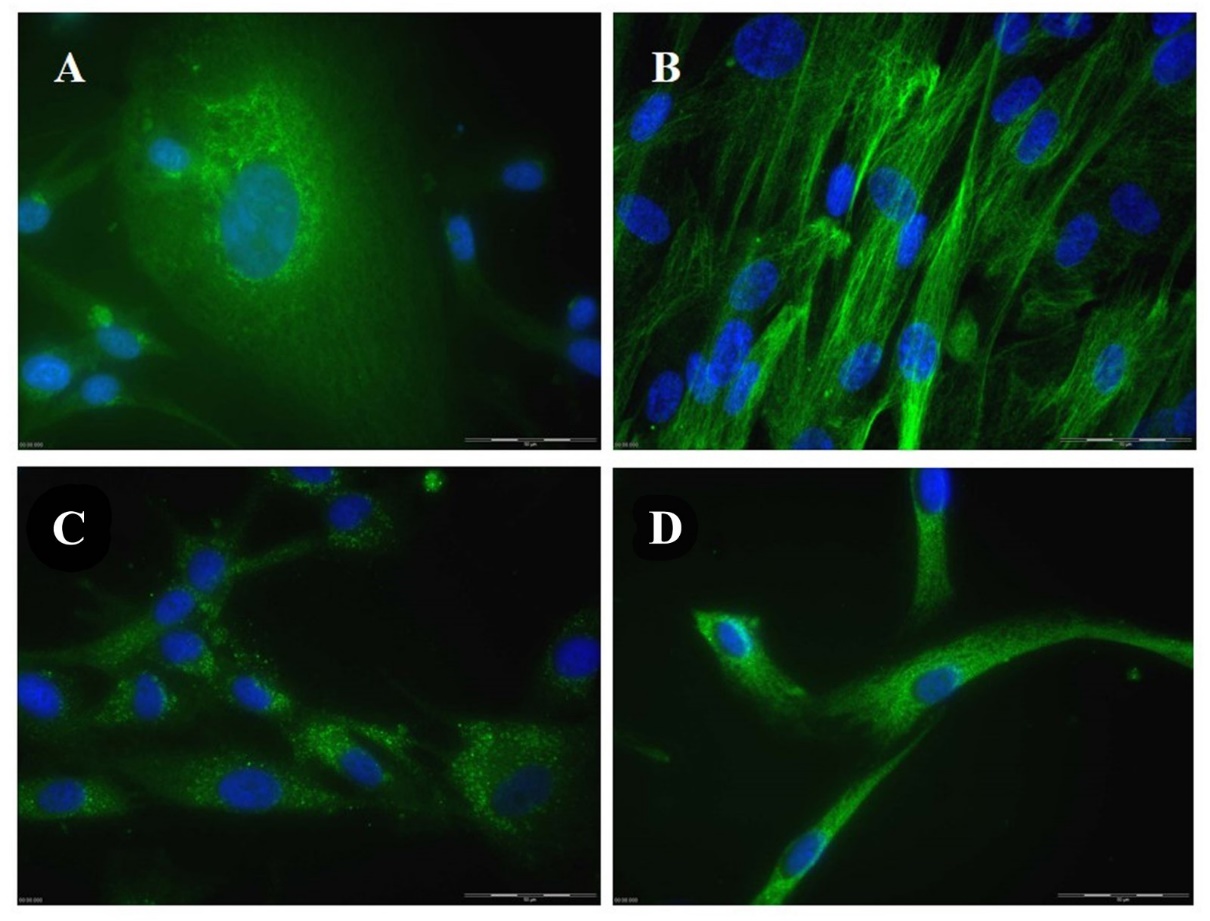


Supplementary Figure S2: Representative micrographs of immunofluorescence analyses in p-WJ-MSCs. Naïve p-WJ-MSCs expressed GATA-4 at perinuclear and cytoplasmatic level (A), nestin at cytoplasmic level (B), connexin-43 (C) and -45 (D) with membrane staining. Magnification: 20x. Scale bar: 100 µm.


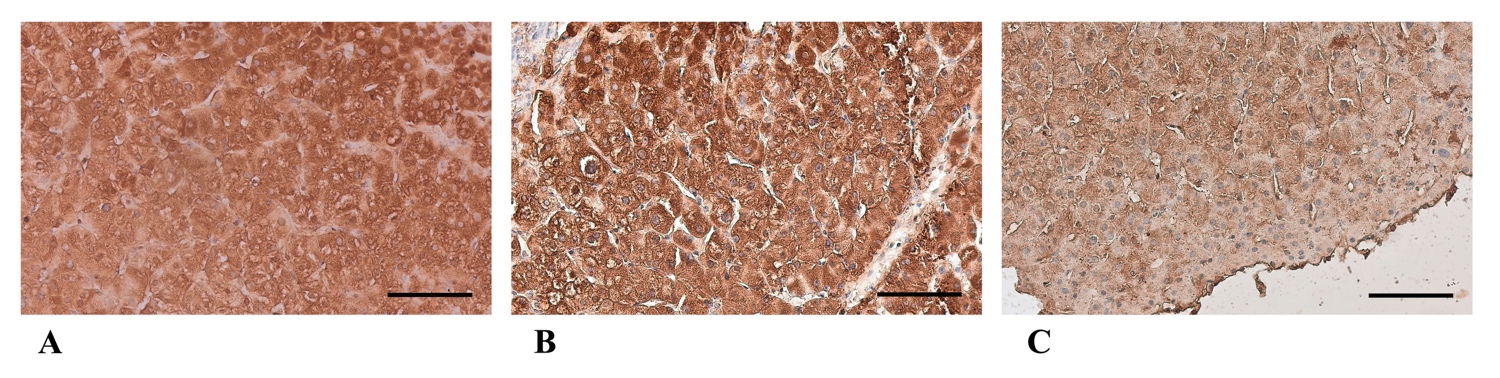


Supplementary Figure S3: Immunohistochemistry analysis for the expression of positive control markers in liver sections. (A) Albumin, (B) cytokeratin-18, and (C) connexin-32. Magnification: 20x. Scale bar: 100µm.


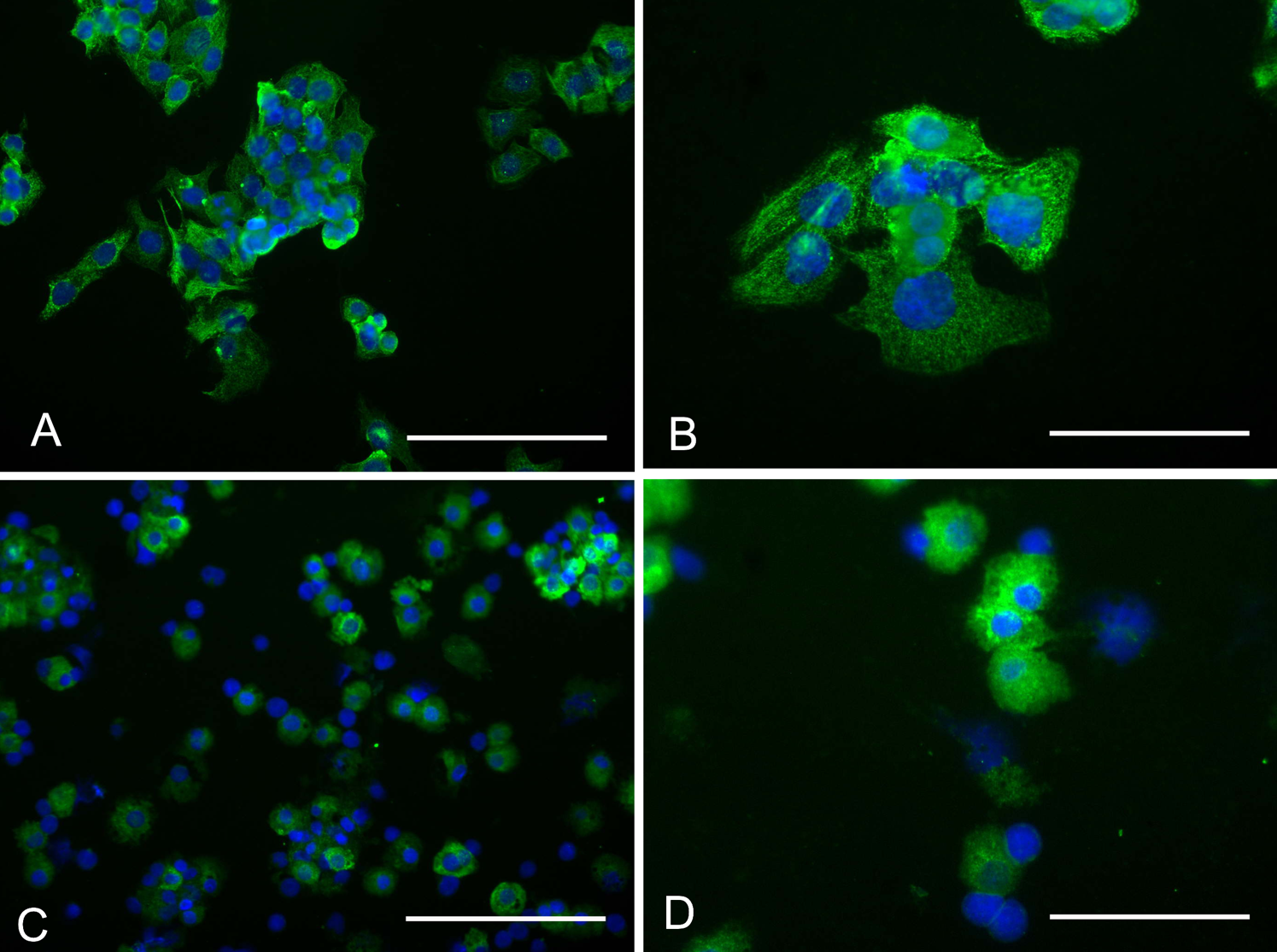


Supplementary Figure S4: Immunofluorescence analysis in HepG2 cells, used as positive control. A, B: albumin expression; C-D: AFP expression. A-C, Magnification: 20X, Scale bar: 100µ; B,D, Magnification: 40X, Scale Bar: 50µ.
